# Allogeneic Bone Marrow–Derived Mesenchymal Stem Cell Safety in Idiopathic Parkinson's Disease

**DOI:** 10.1002/mds.28582

**Published:** 2021-03-27

**Authors:** Mya Schiess, Jessika Suescun, Marie‐Francoise Doursout, Christopher Adams, Charles Green, Jerome G. Saltarrelli, Sean Savitz, Timothy M. Ellmore

**Affiliations:** ^1^ Department of Neurology, McGovern Medical School University of Texas Health Science Center at Houston Houston Texas USA; ^2^ Department of Anesthesiology, McGovern Medical School University of Texas Health Science Center at Houston Houston Texas USA; ^3^ Department of Pediatrics, McGovern Medical School University of Texas Health Science Center at Houston Houston Texas USA; ^4^ Department of Surgery, McGovern Medical School University of Texas Health Science Center at Houston Houston Texas USA; ^5^ Department of Psychology The City College of New York New York City New York USA

**Keywords:** donor‐specific antibodies, neuroinflammation, mesenchymal stem cells, clinical trials, patient safety

## Abstract

**Background:**

Neuroinflammation plays a key role in PD pathogenesis, and allogeneic bone marrow–derived mesenchymal stem cells can be used as an immunomodulatory therapy.

**Objective:**

The objective of this study was to prove the safety and tolerability of intravenous allogeneic bone marrow–derived mesenchymal stem cells in PD patients.

**Methods:**

This was a 12‐month single‐center open‐label dose‐escalation phase 1 study of 20 subjects with mild/moderate PD assigned to a single intravenous infusion of 1 of 4 doses: 1, 3, 6, or 10 × 10^6^ allogeneic bone marrow–derived mesenchymal stem cells/kg, evaluated 3, 12, 24, and 52 weeks postinfusion. Primary outcome safety measures included transfusion reaction, study‐related adverse events, and immunogenic responses. Secondary outcomes included impact on peripheral markers, PD progression, and changes in brain perfusion.

**Results:**

There were no serious adverse reactions related to the infusion and no responses to donor‐specific human leukocyte antigens. Most common treatment‐emergent adverse events were dyskinesias (20%, n = 4) with 1 emergent and 3 exacerbations; and hypertension (20%, n = 4) with 3 transient episodes and 1 requiring medical intervention. One possibly related serious adverse event occurred in a patient with a 4‐year history of lymphocytosis who developed asymptomatic chronic lymphocytic leukemia. Peripheral inflammation markers appear to be reduced at 52 weeks in the highest dose including, tumor necrosis factor‐α (*P* < 0.05), chemokine (C‐C motif) ligand 22 (*P* < 0.05), whereas brain‐derived neurotrophic factor (*P* < 0.05) increased. The highest dose seems to have demonstrated the most significant effect at 52 weeks, reducing the OFF state UPDRS motor, −14.4 (*P* < 0.01), and total, −20.8 (*P* < 0.05), scores.

**Conclusion:**

A single intravenous infusion of allogeneic bone marrow–derived mesenchymal stem cells at doses of 1, 3, 6, or 10 × 10^6^ allogeneic bone marrow–derived mesenchymal stem cells/kg is safe, well tolerated, and not immunogenic in mild/moderate PD patients. © 2021 The Authors. *Movement Disorders* published by Wiley Periodicals LLC on behalf of International Parkinson and Movement Disorder Society

Neurological disorders are the leading cause of disability worldwide, and Parkinson's disease (PD) is the fastest growing. Neurodegeneration in PD is relentlessly progressive, creating a compelling need to find effective and safe disease‐modifying therapies. Considerable evidence supports the critical role of neuroinflammation in the degenerative process,^1^ which is known to be orchestrated by interactions of glial cells, peripheral lymphocytes, proinflammatory cytokines/chemokines, and changes in growth factors.^2^, ^3^, ^4^, ^5^ The inflammatory condition of the parkinsonian neuronal‐glial microenvironment is well described in human postmortem tissue, in vivo models (1‐methyl‐4‐phenyl‐1,2,3,6‐tetrahydropyridine, 6‐hydroxydopamine, rotenone, lipopolysaccharide (LPS), and SNc extracts), and in vitro models. Inflammation also plays a significant role in toxin‐induced and genetic models,^6^, ^7^ and epidemiological studies on the risk‐lowering effects of anti‐inflammatory drug regimens confirm its vital role.^8^, ^9^ Our group has studied the peripheral immune system in PD neurodegeneration by using LPS rat models,^10^, ^11^ glial cells,^12^, ^13^ and patient cerebrospinal fluid and blood.^14^, ^15^ Results from these investigations indicate that an adaptive immune response contributes to progression and supports the rationale for using an immune‐modulatory therapy such as mesenchymal stem cells (MSCs). MSCs have been studied in multiple PD animal models,^16^, ^17^, ^18^ and the potential benefit relies primarily on paracrine actions, exosomal activity, and modulation of host immune cells. Preclinical data on MSC therapy has indicated a positive effect as measured by decreased dopamine (DA) neuron loss, inflammatory cytokine production, microglial activation, and α‐synuclein oligomerization along with increased DA neuronal regeneration.^19^, ^20^, ^21^

The primary objective of this study was to prove safety and feasibility in the first‐of‐its‐kind use of allogeneic bone marrow–derived mesenchymal stem cells (allo‐hMSCs) delivered intravenously in escalating doses to patients with idiopathic PD.

## Methods

### Experimental Design

We conducted a single‐center, open‐label, ascending‐dose‐escalation phase 1 clinical study in patients with mild to moderate PD. Twenty participants were enrolled sequentially into 1 of 4 dose groups and received a single intravenous infusion: group A, 1 × 10^6^ allo‐hMSCs/kg; group B, 3 × 10^6^ allo‐hMSCs/kg; group C, 6 × 10^6^ allo‐hMSCs/kg; and group D, 10 × 10^6^ allo‐hMSCs/kg. Subjects returned for assessments in weeks 3, 12, 24, and 52 after infusion. An Investigational New Drug (IND) was obtained from the US Food and Drug Administration (FDA; #16756). A staggered design was created to maximize safety, which consisted of a 1 + 1 + 1 + 2 schedule for groups A and B, followed by a 2 + 3 for groups C and D, with 30 days between infusions. Doses were based on safety and efficacy literature in various disorders^22^, ^23^, ^24^, ^25^, ^26^, ^27^, ^28^ and the optimal volume necessary to preserve cell quality and viability. The sample size of 20 active participants was based on epidemiological estimates; incidence rates of potential serious adverse events (SAEs) ranged from 5.5/1,000,000 to 14/1000. The design provided 90% confidence intervals for detecting a potential SAE that ranged from 0.2% to 18.3%. The protocol was approved by our institutional review board (UTHealth institutional review board), HSC‐MS‐16‐0026, and registered with clinicaltrials.gov, NCT:02611167. All participants provided written informed consent. The study was conducted according to the International Council for Harmonization Good Clinical Practice guidelines and the principles of the Declaration of Helsinki. An independent data and safety monitoring board (DSMB) monitored the conduct of the trial and subject safety.

### Study Participants

Immunocompetent male and female patients between 45 and 78 years old with mild to moderate PD were recruited after being screened through a questionnaire that included medical history details. Critical enrollment criteria included UK Brain Bank criteria; an OFF‐state Hoehn and Yahr scale (H&Y) score ≤ 3; robust response to dopaminergic therapy, defined as ≥33% reduction of the Unified Parkinson's Disease Rating Scale (UPDRS) motor score in the OFF versus ON state; a stable dose of dopaminergic replacement at baseline (≥60 days); and onset of motor symptoms of ≥4 and ≤10 years. Subjects were excluded if they had features of atypical or secondary parkinsonism; a history of DBS or ablative brain surgery; or a Montreal Cognitive Assessment (MoCA) < 23.

### Outcomes

The primary study objective was to evaluate the safety and tolerability of an intravenous infusion of bone marrow–derived allo‐hMSCs. Exploratory secondary objectives involved assessing relevant biomarkers for mechanism of action and clinical assessments of PD progression.

Safety and tolerability were evaluated by the presence of treatment‐emergent adverse events (TEAEs), laboratory tests (complete blood count, complete metabolic panel, glomerular filtration rate, international normalized ratio, prothrombin time, and partial thromboplastin time), suicidal ideation, physical and neurological examinations, and changes in panel reactive antibody (PRA). Exploratory clinical assessments involved 4 different domains (cognition and behavioral changes, motor function, disability) and quality of life and included the UPDRS/MDS‐UPDRS; H&Y; Timed‐Up‐and‐Go (TUG); Modified Schwab and England Activities of Daily Living Scale (ADL); 39‐item Parkinson's Disease Questionnaire (PDQ‐39); MoCA; the Columbia Suicide Severity Rating Scale; and the 40‐item University of Pennsylvania Smell Identification Test (UPSIT). All the motor assessments were done by a movement disorders specialist in the conventional OFF state, defined as being OFF PD medicines 12 hours prior to the examination. Patients were required to maintain their baseline dopaminergic regimen for 24 weeks postinfusion.

### Allogeneic Human Mesenchymal Stem Cells

Bone marrow extraction from a healthy donor was obtained by aspiration under local anesthesia. Testing was performed using FDA‐approved licensed kits by Gulf Coast Regional Blood Center. MSCs were expanded using a Terumo Quantum Bioreactor^29^ by the Center for Cell and Gene Therapy of Baylor College of Medicine under current Good Manufacturing Practices designated by the FDA. The total quantity of allo‐hMSCs was reached in 3 passages. Thawing was initiated on infusion day, and allo‐hMSCs were aliquoted into a 250‐mL transfer pack with 5% buminate. Release tests were performed on the pooled cells (purity, viability, cell dose, and microbiological testing).

### Peripheral Markers

Serum samples obtained at baseline and after 3, 12, 24, and 52 weeks were analyzed for chemokines, cytokines, and growth factor concentrations using a Millipore Milliplex MAP 37‐plex human cytokine/chemokine panel and a Milliplex MAP Human Neurodegenerative Disease Magnetic Bead Panel 3 (Millipore, Billerica, MA). Precisely, tumor necrosis factor (TNF)–α, CXCL10/IP‐10, chemokine (C‐C motif) ligand (CCL) 2/monocyte chemoattractant protein‐1, CCL11/Eotaxin, CCL22/MDC, and brain‐derived neurotrophic factor (BDNF) were measured. Plates were analyzed on a Luminex 200 equipped with xPONENT 3.1 software (Luminex, Austin, TX).

### Neuroimaging

Participants underwent MRI scanning in the OFF state at baseline and 24 weeks postinfusion on a 3‐Tesla Philips Ingenia using a 32‐channel head coil. Imaging sessions included a high‐resolution T1‐weighted anatomical (repetition time/echo time [TR/TE], 8.1/3.7 milliseconds; flip angle, 6 degrees; matrix size, 256 × 256; field of view, 256 mm; slice thickness, 1.0 mm, sagittal acquisition) and a pseudo‐continuous arterial spin labeling (pCASL) sequence (TR/TE, 4550/15.8 milliseconds; flip angle, 90 degrees; slice thickness, 5 mm; 240‐mm field of view; slice gap, 1·0 mm; voxel resolution, 1.875 × 1.875 × 6 mm^3^; 30 dynamic signal averages, with a labeling duration of 1650 milliseconds and a postlabeling delay of 1600 milliseconds) used to measure noncontrast brain perfusion. Average perfusion in 8 basal ganglia regions of 2 hemispheres was compared using the multicontrast PD25 atlas.^30^ The PD25 T1 volume with 1‐mm resolution was coregistered to each subject's native‐space skull‐stripped T1 anatomical volume in AFNI.^31^

### Panel Reactive Antibody

Human leukocyte antigen (HLA) level against class I and class I antigens was assessed using One Lambda (West Hills, CA) LABScreen Single Antigen Bead Assay evaluated on either a Luminex 200 or Luminex LabScan 3D. This information was compared against a national database to determine the PRA as well as being interrogated against donor HLA typing to determine the presence of donor‐specific antibodies (DSA). PRA is based on antibody frequency (not strength) and is used to determine the percent of the population the patient will be positive against.

### Statistical Analysis

Demographic characteristics and safety data were summarized using descriptive statistics. Peripheral markers and rating scales were analyzed per dose group by 1‐way, repeated‐measures analysis of variance (ANOVA) evaluating the effect of time on each peripheral marker and outcome scale. Statistically reliable omnibus (*F* tests) finds resulted in post hoc follow‐up tests using dependent‐sample *t* tests to evaluate each time against its respective baseline. Given the small sample size, the early phase of investigation and desire to avoid a potential type II error, we treated such comparisons as hypothesis‐generating using *P* ≤ 0.05 as a threshold for statistical testing. The pCASL regions of interest values were analyzed using a 3‐way ANOVA (region × time × cohort) with a Tukey multiple‐comparisons correction. An additional voxel‐wise analysis was performed in ExploreASL^32^ to examine the location of perfusion changes within the basal ganglia regions. All analyses were done using Prism 5.0 (Graph Pad Software, Inc., San Diego, CA).

## Results

### Study Population

The study prescreened 152 candidates and screened 21. One patient was a screen failure because of renal insufficiency, and the remaining 20 were sequentially enrolled in 1 of 4 groups 1, 3, 6, or 10 × 10^6^ allo‐hMSCs/kg of body weight, with n = 5 per group. Baseline characteristics appeared similar across groups except for cognition (Table 1). MoCA scores were significantly different because of the low mean for group C (*P* < 0.05). Subjects had a mean age of 66 years, H&Y of 2 or 2.5 (65%), disease duration between 4 and 6 years (65%), and mean total UPDRS score of 50.4. All but 1 participant used dopaminergic medications before baseline evaluation.

**TABLE 1:** Demographics and baseline characteristics

Group	A	B	C	D
Dose of MSC/kg	1 × 10^6^	3 × 10^6^	6 × 10^6^	10 × 10^6^
Number of subjects	5	5	5	5
Sex, female:male ratio	3:2	2:3	1:4	3:2
Age (y)	65.4 (9.5)	68.2 (6.4)	65.8 (7.9)	66.4 (5.9)
Disease duration (y)	5.9 (1.5)	4.7 (3.0)	5.8 (1.5)	5.6 (0.9)
Handedness, right:left ratio	5:0	5:0	4:1	3:2
Race(white, Hispanic, Asian, African American, Native American)	White, 4 (80%)Asian, 1 (20%)	White, 4 (80%)African American, 1 (20%)	White, 3 (60%)Hispanic, 2 (40%)	White, 4 (80%)Hispanic, 1(20%)
MoCA	27 (2.0)	28 (1.7)	24.6 (0.9)	26.6 (1.7)
UPSIT 40‐item test	25.8 (7.1)	15.4 (6.7)	22.4 (6.5)	19.4 (6.3)
UPDRS‐M^a^	32.8 (8.7)	36.2 (18.1)	30.2 (13.7)	36.0 (11.5)
UPDRS‐T^a^	52.8 (18.9)	48.8 (23.4)	47 (17.2)	53.8 (13.0)
MDS‐UPDRS‐M^a^	35 (11.4)	39.6 (20.9)	34.2 (14.0)	45 (13.1)
MDS‐UPDRS‐T^a^	67.6 (24.0)	57.2 (29.7)	67.8 (19.9)	78.2 (15.4)
H&Y^a^	1.7 (0.7)	2 (0.7)	2.3 (0.4)	2.4 (0.5)
TUG,^a^ s	17.8 (7.2)	19.7 (5.6)	18.8 (6.6)	15.7 (3.3)
LEDD	711 (403.9)	797.4 (288.5)	438.2 (358.3)	674.6 (203.9)
Levodopa challenge, % improvement	66.2 (14.6)	69.8 (13.1)	64.8 (18.6)	77.2 (7.3)

Data are mean (SD) or n (%).

^a^ Performed in the conventional OFF state (≥12 hours without PD meds).

Levodopa challenge, percentage of improvement in the UPDRS‐M in the ON state versus the OFF state.

### Primary Outcome: Safety

All 20 subjects received a single intravenous infusion and were monitored for 24 hours postinfusion. Three patients reported TEAEs, 1 phlebitis, 1 antecubital fossa hematoma, and 1 headache (Table 2). The first patient had 3 cm superficial phlebitis grade 2 infusion‐related allergic reactions (National Cancer Institute‐Common Terminology Criteria for Adverse Events (NCI‐CTCAE) criteria) that required local medical management. The other 2 patients had mild symptoms and did not require any treatment. All patients left the UTHealth research unit with full resolution of their symptoms. In subsequent follow‐up, 50% of the patients reported TEAEs; most of them were mild and transient, with dyskinesia (20%, n = 4) and hypertension (20%, n = 4) as the most common. Three of the patients experienced exacerbated dyskinetic activity (preexisting dyskinesias documented at baseline), 1 at 3 weeks and the other 2 at 24 weeks postinfusion, requiring levodopa reduction for resolution; the remaining newly emergent dyskinesia case presented at 24 weeks and resolved without intervention. Three of the four hypertension cases were reported between 3 and 12 weeks after infusion and were followed by their primary care physicians, determined to be transient and required no intervention. One patient was diagnosed with stage 2 hypertension that emerged 24 weeks postinfusion and persisted through the 52‐week follow‐up period necessitating medical management.

**TABLE 2:** Treatment‐emergent adverse events (TEAEs)

Body system	Adverse reaction	Group A n = 5 (100%)	Group B n = 5 (100%)	Group C n = 5 (100%)	Group D n = 5 (100%)	AE relationship to MSC
Cardiovascular	Hypertension	2 (40%)	2 (40%)	0	0	Unlikely related
	Phlebitis^a^	0	0	0	1 (20%)	Related
	Hematoma^a^	0	0	1 (20%)	0	Related
Gastrointestinal	Nausea^b^	1 (20%)	2 (40%)	0	0	Possibly related
Neurologic	Headache^a^	0	1 (20%)	0	0	Possibly related
	Dyskinesia	1 (20%)	0	2 (40%)	1 (20%)	Probably related
Hematology	CLL	0	0	1 (20%)	0	Possibly related
Laboratory	↓Lymphocytes^b^	3 (60%)	2 (40%)	0	1 (20%)	Possibly related
	↑Basophils^b^	1 (20%)	1 (20%)	1 (20%)	1 (20%)	Possibly related

Data show n (%) per dose group.

^a^ There were 3 AEs during the infusion procedure.

^b^ Mild and transient. Four patients required management, 1 for hypertension and 3 for exacerbation of dyskinesias.

CLL, chronic lymphocytic leukemia.

There was 1 SAE during the study. Eight months after allo‐hMSC infusion, 1 patient in group B, with a 4‐year history of lymphocytosis, was diagnosed with asymptomatic chronic lymphocytic leukemia (CLL). At the time of enrollment, the patient did not disclose a previous history of lymphocytosis, was asymptomatic, and had a slight increase in lymphocyte percentage. The patient's lymphocyte percentage remained out of range during the entire study, with normal white blood cell and lymphocyte absolute numbers. Based on the lack of literature on this adverse event in studies with allo‐hMSCs, our DSMB decided to label the SAE as possibly related. At the end of the study, the patient remained asymptomatic, has not received chemo‐reductive therapy, and is under scheduled monitoring.

Laboratory assessment showed a transient decrease in lymphocytes in 30% of the patients (n = 6) and a transient increase in basophils in 20% of the patients (n = 4). These changes did not last more than 3 months and were not related to any specific disease process.

Suicidal ideation did not emerge during the study. There was no relationship between the incidence of adverse events and dose. No trends emerged between the dose groups from physical or neurological examinations.

### Panel Reactive Antibody

Serum samples were compared with baseline 3, 12, 24, and 52 weeks postinfusion. Of the 20 patients, 17 demonstrated the presence of anti‐HLA antibodies, with 14 possessing anti‐HLA antibodies with identical specificities or fewer throughout the study, with no DSA. Of the 3 samples that contained DSA postinfusion, only 2 had de novo DSA. One of the 2, with de novo DSA presented with an upper respiratory infection, with the sample containing suspected DSA being collected during the late stage of infection with no detectable DSA in subsequent samples. The final patient with suspected de novo DSA exhibited diarrhea of unknown origin around the time of sample collection. DSA was not present in subsequent samples.

### Peripheral Responses

**FIG. 1:**
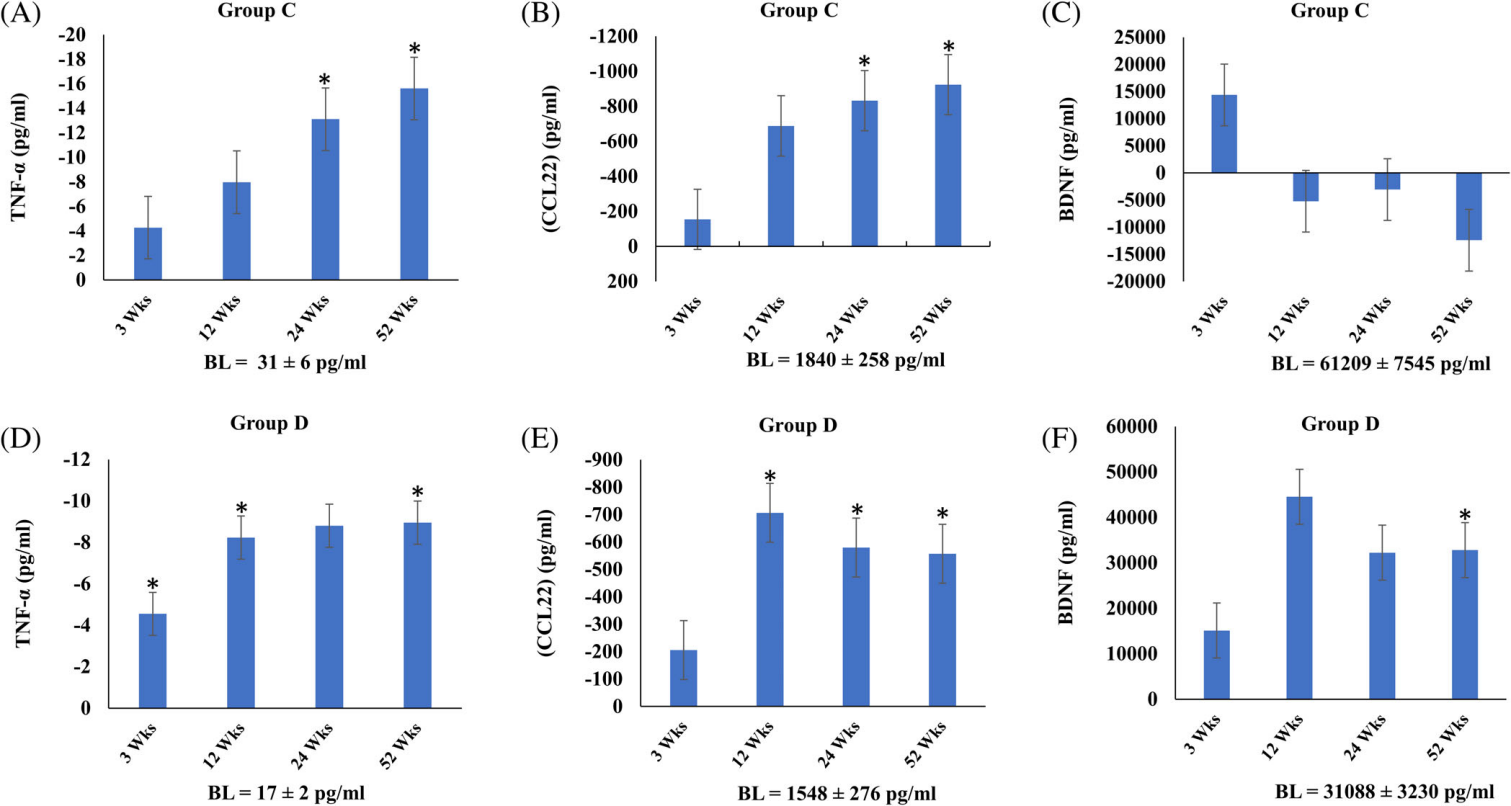
Changes in cytokines, chemokines, and growth factor after allo‐hMSC infusion. Mean ± SEM change compared with baseline (BL) values for TNF‐α, CCL22, and BDNF for group C (**A–C**) and group D (**E–G**). *P < 0.05.

Overall, our data showed downregulation of inflammatory cytokines paired with an increase in BDNF occurring in the higher‐dose groups C and D (Fig. 1 *)*. TNF‐α, a proinflammatory cytokine, decreased at 24 and 52 weeks in group C by 42% and 50%, respectively, and at 3, 12, and 52 weeks in group D by 26%, 48%, and 54%, respectively (*P* < 0.05). CCL22, which displays chemotactic activity for monocytes, dendritic cells, natural killer cells, and T lymphocytes, decreased at 24 and 52 weeks in group C by 45% and 50%, respectively, (*P* < 0.05) and by 46%, 37%, and 36% at 12, 24, and 52 weeks, respectivey, in group D (*P* < 0.05). Last, BDNF, a neurotrophic factor that modulates synaptic plasticity and promotes neuronal survival, increased by 50% at 52 weeks in group D (*P* < 0.05). There was no significant change in TNF‐α, CCL22, and BDNF for groups A and B (data shown in Supplemental Table S1). CXCL10, CCL2, and CCL11 showed no consistent response trend in any of the 4 groups.

### Pseudo‐Continuous Arterial Spin

**FIG. 2:**
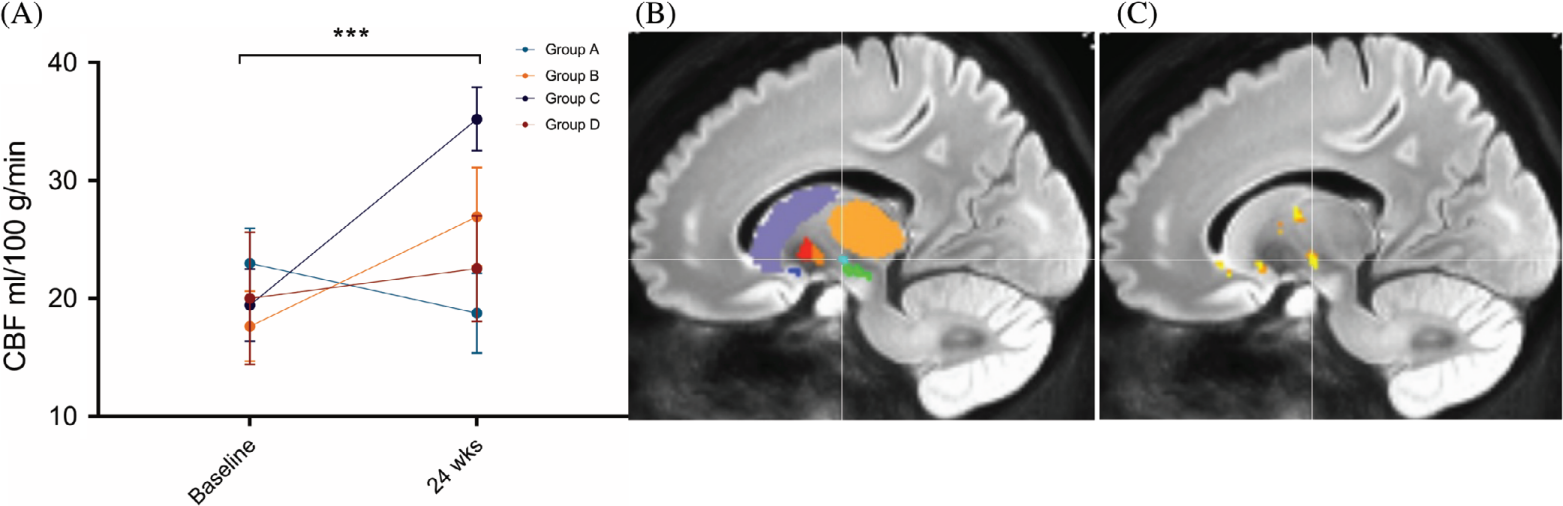
Perfusion changes. (**A**) Mean ± SEM CBF change in STN by dosage group and time. ***P < 0.001. (**B**) Sagittal view with atlas ROIs and crosshair on STN (cyan) with SN (green) located caudally. (**C**) Voxel‐wise analysis confirms cluster of perfusion difference in STN (MNI, −14, −11, −6).

Pseudo‐continuous arterial spin showed a significant main effect of time (*P* < 0.001) and region (*P* < 0.001), but no significant main effect of hemisphere (*P* = 0.401) or cohort (*P* = 0.088). Perfusion increased overall from baseline to 24 weeks postinfusion in all basal ganglia structures (mean difference ± standard error, 2.980 ± 0.590 mL·min/100 g; Tukey's *t* = 5.049, *P* < 0.001). Post hoc time‐by‐region comparisons revealed the most significant increase in perfusion as a function of time after the allo‐hMSC infusion was in the subthalamic nucleus (STN; mean difference ± standard error, 5.844 ± 1.669 mL·min/100 g; Tukey's *t* = 3.501, *P* = 0.042; Fig. 2).

### Rating Scales

**FIG. 3:**
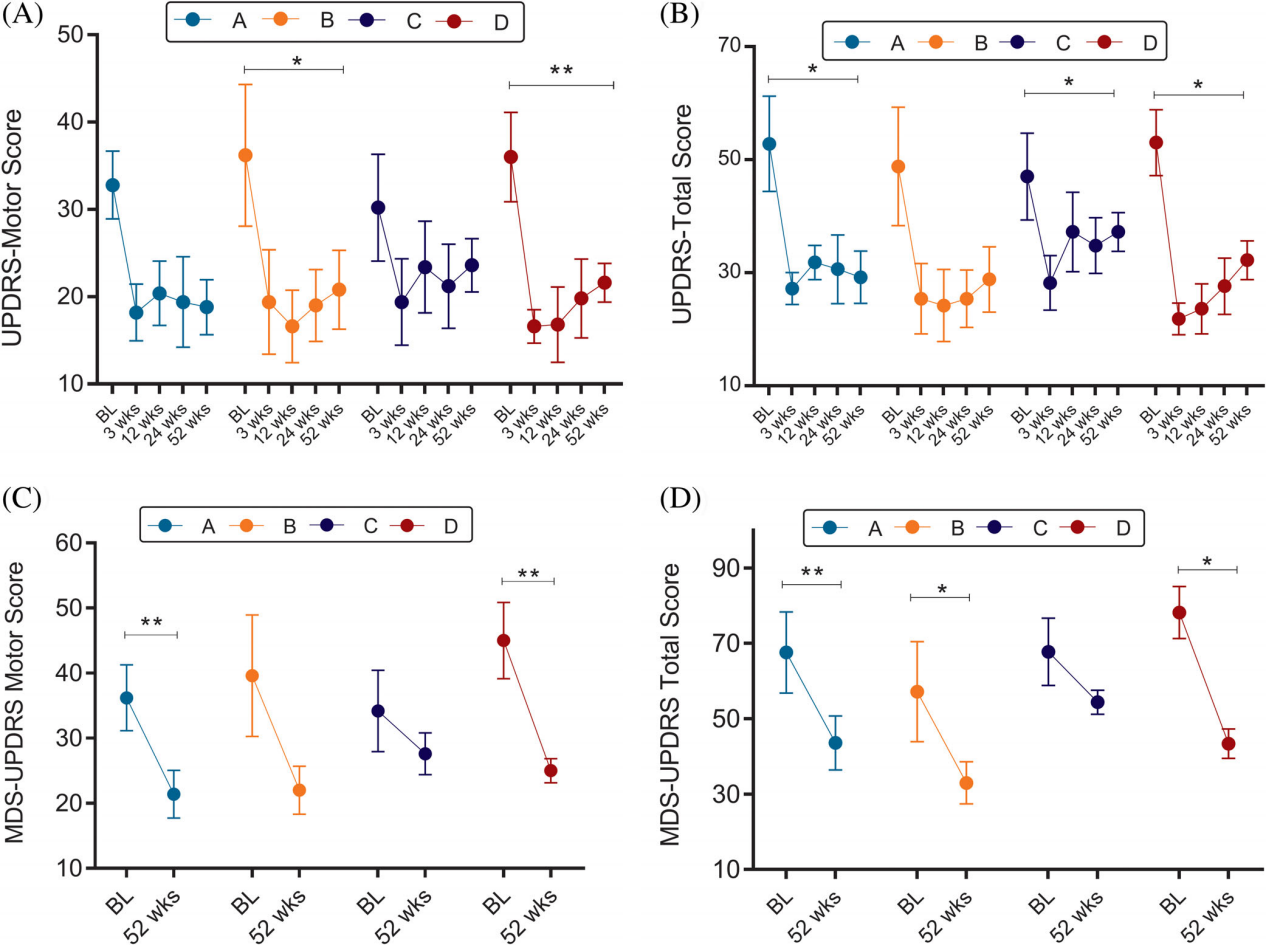
UPDRS and MDS‐UPDRS total and motor changes per dose group after allo‐hMSC infusion. Depicts rating scales mean ± SEM changes per group. (**A**) UPDRS motor score. (**B**) UPDRS total score. (**C**) MDS‐UPDRS motor score. (**D**) MDS‐UPDRS total score. *P < 0.05, **P < 0.01.

All patients sustained motor improvement when tested in the OFF state (Fig. 3A–D). Except for 1 patient who developed dyskinesias 3 weeks postinfusion and required reduction in dopamine, all patients maintained their baseline dopaminergic regimen for 24 weeks postinfusion. The highest dose (10 × 10^6^ allo‐hMSCs/kg, group D) had the most significant effect on reducing the UPDRS total, UPDRS motor, and H&Y scores. At 52 weeks, group D sustained a mean ± SD reduction of 14.4 ± 8.6 in UPDRS motor (*P* < 0.01), 20.8 ± 12.4 in UPDRS total (*P* < 0.05), and 0.5 ± 0.3 in H&Y (*P* < 0.05) scores. This decrease was also seen in the patients who developed dyskinesia as an AE. MDS‐UPDRS was measured only at baseline and week 52 postinfusion. The most substantial reduction in MDS‐UPDRS total (mean ± SD, 34.8 ± 17.5; *P* < 0.05) and MDS‐UPDRS motor (mean ± SD, 20 ± 9.7; *P* < 0.01) scores were also seen in group D. This was the case for self‐reported ADL scores as well, which were significantly increased in weeks 3, 12, and 24 in group D (*P* < 0.01). At 52 weeks, all groups had a clinically significant reduction in their PDQ‐39 scores; the most considerable reduction was 18.8 points in group D (not statistically significant). There was no significant change for the TUG, UPSIT, and MoCA scores.

## Discussion

To our knowledge, this is the first study to demonstrate that a single intravenous infusion of allo‐hMSCs is safe, well tolerated, and not immunogenic at doses that range from 1 to 10 × 10^6^ allo‐hMSCs/kg in subjects with mild to moderate PD. Our pilot study recruited a diverse group of patients who were sequentially enrolled into 1 of 4 escalated‐dose cohorts. All patients received 1 infusion of their assigned dose without any severe adverse events occurring during the infusion or within the first 24 hours. Over a 52‐week follow‐up, the most common side effects were dyskinesias (20%, n = 4) and hypertension (20%, n = 4). Three of the dyskinetic patients required levodopa reduction, suggesting a possible change in sensitivity to their prior dopamine regimen, supported by the resolution of dyskinesias after levodopa reduction. Future studies will need to monitor the emergence or exacerbation of dyskinesias post‐allo‐hMSC infusions to explain the mechanism underlying this phenomenon.

In addition, our study used a 5% buminate medium as part of the allo‐hMSC infusion, which can lead to a transient blood pressure increase for up to 24 hours because of a rise in oncotic pressure.^33^ Four patients developed hypertension between 3 and 24 weeks postinfusion, which makes it unlikely to be related to the medium, with 3 of them transient and not requiring any therapy. One patient developed stage 2 hypertension 6 months postinfusion requiring medical treatment. During the study there was 1 SAE, defined by our DSMB as possibly related based on the absence of literature on allo‐hMSCs related to this specific event. One patient with a 4‐year history of lymphocytosis was diagnosed with CLL 8 months after the infusion. As a result of the low disease burden and given that the patient was asymptomatic, the treating oncologist did not recommend any cytoreductive therapy. The patient remained asymptomatic at the end of the study and continues to be monitored. Future studies should carefully monitor for lymphocytosis, which should be considered an exclusion criterion.

Undifferentiated MSCs are reported to express no major histocompatibility complex class II and low levels of major histocompatibility complex class I and costimulatory molecules. The cellular microenvironment into which they are transferred can stimulate maturation, leading to a deleterious alloimmune response in the recipient. In this study, 70% of the population had preformed anti‐HLA antibodies prior to infusion and either maintained a level of sensitization or had a reduction throughout the study. Fifteen percent were not sensitized against HLA antigens, at any point, whereas another 15% demonstrated anti‐HLA sensitization with DSA, which resolved during the course of the study. Based on these data, a single infusion of mesenchymal stem cells did not lead to the development of anti‐HLA antibodies. In future studies with repeated doses, DSA response should be monitored carefully and should be used as a decisive safety tool for infusion continuation.

Allo‐hMSC effects are likely based on immunomodulation and neurotrophic support, which potentially can be achieved through peripheral administration. Compared with other routes of delivery, the intravenous route is the less invasive. Multiple studies have proven that allo‐hMSC quality hinges not only on the culture methods used but also on the donor profile (age, body mass index, genetic traits, and medical history). Isolated MSCs from elderly and/or obese donors have decreased biological activity.^34^ To date, there have been numerous studies of the effects of intravenous MSC therapy on other neurodegenerative diseases, including multiple system atrophy,^35^ progressive supranuclear palsy,^36^ multiple sclerosis,^37^ and amyotrophic lateral sclerosis (ALS),^38^ all of which revealed safety and indicated some evidence of efficacy. This knowledge, along with the lower immunogenicity of MSCs, supported our choice of using allo‐hMSCs from young, healthy donors as a potentially effective and viable option for PD treatment.

The potential therapeutic benefit of MSC therapy relies primarily on paracrine actions.^39^, ^40^ In this study, multiple peripheral markers were assessed to elucidate possible mechanisms of action. Our findings suggest a reduction in serum levels of TNF‐α and CCL22, with an increase in BDNF. There was an anti‐inflammatory effect that appeared to be more robust in the higher doses of groups C and D and may be related to a potential mechanism of action. High levels of TNF‐α and CCL2 and low levels of BDNF have been previously reported in PD patients.^41^, ^42^, ^43^

Moreover, high levels of TNF‐α correlate with worse motor scores.^44^ Previous animal studies have shown that intravenous MSCs can reduce TNF‐α in the substantia nigra^45^ and that systemic deficiency of TNF‐α is associated with improvement.^46^ Similar to our study, in 2 clinical trials using intravenous MSCs, CCL22 was significantly decreased at 12 months in autism spectrum disorder^47^ and at 2 weeks after infusion in an ALS study.^48^ We hypothesized that these peripheral changes could lead to an anti‐inflammatory microglial phenotype, which in turn could enhance neuronal survival and promote angiogenesis, consistent with the finding of increased basal ganglia perfusion.

Furthermore, we found no evidence for hemispheric differences in perfusion. Although the most significant increase occurred in cohort C, there was no significant main effect of the overall dose groups. There was an increase in STN perfusion. The STN plays an essential role in PD symptomatology, especially in direct–indirect pathway imbalance. Studies reporting resting cerebral blood flow (CBF) perfusion, as opposed to resting connectivity of STN, are limited. However, a recent report of ASL‐derived CBF values found no significant differences between early PD patients and controls, although there was a trend for increased perfusion in PD that did not survive multiple‐comparison corrections.^49^ This may be concordant with increased neuronal activity, but whether it corresponds to regularization of an irregular firing pattern is unclear.

The clinical assessments (done in the conventional OFF state) demonstrated changes in motor and nonmotor symptoms starting 3 weeks postinfusion and persisted to the end of the study. For most patients, rating scores slowly increased over time after the 12‐week postinfusion. However, both the total and motor subscales of the UPDRS and MDS‐UPDRS never returned to baseline levels, with group D having the most significant effect. Motor improvement over the 52 weeks is not expected in the natural progression of PD^50^, ^51^ and supports the need for a phase 2 clinical trial to test the efficacy of allo‐hMSCs as a disease‐modifying therapy in Parkinson's disease.

Study limitations include the small number of patients, lack of genetic testing and subtyping as part of the dose assignment process, and the lack of a placebo group. Differences in genetic makeup and phenotypes might impact treatment response and should be considered in further trials. In addition, 16% of the UPDRS motor improvement could be attributed to the placebo effect; however, this effect rarely lasts more than 6 months.^52^ Last, our current tools for assessing disease severity may be limited, and reliance on estimates of disease duration based on time of diagnosis may lack accuracy because motor symptoms may have started long before an official diagnosis.

In conclusion, this study demonstrated that a single infusion of allogeneic MSCs ranging from 1 to 10 × 10^6^ intravenous allo‐hMSCs/kg was safe, well tolerated, and not immunogenic in patients with mild‐ to moderate‐stage Parkinson's disease. Based on our secondary outcomes, our study identified a possible dose that had the greatest effect on clinical progression. This information supports moving forward to a phase 2 randomized, placebo‐controlled efficacy trial using allo‐hMSCs in a larger population of well‐defined Parkinson's disease patients.

## Data Safety Monitoring Board

Toby Yaltho, MD (Movement Disorder Neurologist, Houston Methodist Sugar Land Hospital, Houston, TX), Joshua Samuels, MD (Professor, Department of Pediatrics, Evidence‐Based Medicine, Nephrology Fellowship Program Director, University of Texas Health Science Center at Houston, Houston, TX), and Barbara Tilley, PhD (Professor, Department of Biostatistics, Lorne D. Bain Distinguished Professorship in Public Health and Medicine, University of Texas Health Science Center at Houston, Houston, TX).

## Data Sharing

Deidentified data collected for the study, including individual participant data, a data dictionary defining each field in the set, and the study protocol, will be made available through REDcap to researchers who provide a methodologically sound proposal, beginning 3 months and ending 36 months following article publication. Proposals should be directed to: jessika.s.ocampo@uth.tmc.edu.

## Author Roles

M.S. led the study. M.S. and J.S. designed and conceptualized the study and drafted the manuscript for intellectual content. M.S. was responsible for patient recruitment, clinical assessment, and study oversight. J.S. was responsible for managing the trial and performed data cleaning. C.A. and M.F.D. were responsible for the peripheral immune analysis and interpretation. T.E. was responsible for imaging analysis and interpretation. J.G.S. was responsible for panel reactive antibody analysis and interpretation. C.A. was responsible for data entry. J.S., T.E., M.F.D., C.G., and J.G.S. were responsible for data analysis. All authors interpreted the data. S.S. was responsible for a critical review of the manuscript. All authors read and edited the drafts of the manuscript and approved the final version.

## Supporting information

**Table S1.** Supporting information
