# Supplementary material for: Allogeneic Bone Marrow–Derived Mesenchymal Stem Cell Safety in Idiopathic Parkinson's Disease
Source: Mov Disord. 2021 Mar 27;36(8):1825–34. doi: 10.1002/mds.28582 (PMC8451899; doi:10.1002/mds.28582)
Supplement: Supplementary file 1 — Table S1. Supporting information [file MDS-36-1825-s001.docx]

| **Supplemental Table 1.** | | | | | | | |
| --- | --- | --- | --- | --- | --- | --- | --- |
| **Baseline values for** **Cytokines, Chemokines, and growth factor (Mean ± SE)** | | | | | | | |
| **Groups** | | **TNF-α (pg/ml)** | | **CCL22 (pg/ml)** | | **BDNF (pg/ml)** | |
| **A** | | 51±44 | | 727±142 | | 38599±4369 | |
| **B** | | 13±5 | | 831±115 | | 32831±3668 | |
| **C** | | 31±6 | | 1840±258 | | 61209±7545 | |
| **D** | | 17±2 | | 1548±276 | | 31088±3230 | |
| **Changes in Cytokines, Chemokines, and growth factor after MSC infusion.** (∆±**SE** ) | | | | | | | |
| **Groups** | **Weeks**  (Comparison to baseline) | | **TNF-α (pg/ml)** | | **CCL22 (pg/ml)** | | **BDNF (pg/ml)** |
| **A** | 3 | | 11.69±5.88 | | 2.35±104.55 | | -1726±3744 |
|  | 12 | | -4.62±5.58 | | -44.73±112.11 | | 3448±2309 |
|  | 24 | | 13.68±11.55 | | -96.84±58.09 | | -3100±2533 |
|  | 52 | | 5.35±2.88 | | 8.94±91.49 | | 20372±11066 |
| **B** | 3 | | -1.75±1.45 | | 2.22±67.59 | | 10881±11447 |
|  | 12 | | -0.65±2.12 | | 112.20±84.04 | | 29468±13671 |
|  | 24 | | -0.65±3.47 | | 122.30±69.91 | | 14672±8334 |
|  | 52 | | 0.62±7.10 | | 89.87±66.47 | | 33830±12639 |
| **C** | 3 | | -4.27±2.98 | | -154.46±184.02 | | 14380±15612 |
|  | 12 | | -7.97±4.14 | | -688.20±303.45 | | -5229±8593 |
|  | 24 | | -13.11±4.76 * | | -832.16±275.07 * | | -3071±10953 |
|  | 52 | | -15.62±4.93 * | | -923.99±253.10 * | | -12403±14230 |
| **D** | 3 | | -4.55±1.61 * | | -205.42±209.45 | | 15120±7113 |
|  | 12 | | -8.23±3.09 * | | -706.31±208.47 * | | 44526±20652 |
|  | 24 | | -8.80±3.05 | | -579.61±195.24 * | | 32230±19478 |
|  | 52 | | -8.95±3.09 * | | -556.90±198.14 * | | 32808±12120 * |
| Data shows baseline values and descriptive statistics for differences and paired t-test per dose group. Effects of MSC on peripheral Tumor Necrosis Factor-alpha; C-C motif chemokine 22; Brain-derived neurotrophic factor. *p<0.05. | | | | | | | |
